# Diagnostic and Prognostic Value of CA19-9 in Pancreatic Tumors: A Retrospective Study

**DOI:** 10.7759/cureus.104809

**Published:** 2026-03-07

**Authors:** Feras A Almbaidin, Mohammad E Alduham, Mo'taz F Naffa', Asia S Aledwan, Alaa A Alzubi, Awad M Jarrar, Zaid M Al Hassan

**Affiliations:** 1 Surgery, Jordanian Royal Medical Services, Amman, JOR

**Keywords:** bilirubin, ca19-9, jaundice, pancreatic ductal adenocarcinoma, postoperative biomarker, resection, survival

## Abstract

Background: Carbohydrate antigen 19-9 (CA19-9) is a widely used biomarker for pancreatic cancer, but the interpretation of its level is confounded by false positives in patients with cholestasis and false negatives in Lewis antigen-negative patients. The aim of the study was to investigate the diagnostic accuracy of CA19-9 in differentiating malignant from benign pancreatic lesions and its prognostic significance following tumor resection.

Methods: We retrospectively reviewed the data of 81 consecutive pancreatic resections performed at Al-Latron Military Hospital, a tertiary center in Amman, Jordan, from January 2016 through June 2024. Preoperative CA19-9 levels, clinical variables, and pathology findings were collected. Diagnostic discriminability was assessed via receiver operating characteristic (ROC) analysis. Multivariable logistic regression was used to assess whether log10(CA19-9) and jaundice could independently predict malignancy. Overall survival (OS) and progression-free survival (PFS) were estimated by Kaplan-Meier survival analysis, and the corresponding curves were compared with log-rank tests, including those of patients stratified a priori by CA19-9 level (<=37 vs. >37 U/mL).

Results: CA19-9 values were available for 73/81 patients (59 with malignant lesions, 14 with benign lesions). Preoperative CA19-9 levels were higher in malignant lesions than in benign lesions (median 86 vs. 12 U/mL; p=0.008). The area under the curve (AUC) was 0.82 (95% CI 0.71-0.90), indicating moderate-to-good discriminative ability. A CA19-9 level of 37 U/mL achieved a sensitivity of 62% (95% CI 48%-74%) and a specificity of 89% (95% CI 62%-98%) in discriminating malignant from benign lesions. In the multivariable analysis, log10(CA19-9) independently predicted malignancy (OR 3.97; 95% CI 1.2-13.0; p=0.02), whereas jaundice did not (p=0.37). After a median follow-up of 24 months, the one- and three-year OS rates for patients with malignant lesions were 93.7% and 81.0%, respectively; median OS was not reached, with 12 death events observed during follow-up. Patients with benign lesions had a 100% survival rate at the last follow-up (p=0.03). Among patients with malignant lesions, OS was numerically greater for CA19-9 ≤ 37 vs. >37 U/mL, but the difference was not statistically significant (p=0.15). Among patients with available postoperative measurements, postoperative CA19-9 elevation appeared to accompany early recurrence; however, postoperative sampling was not standardized, and this observation is descriptive only.

Conclusions: CA19-9 is a useful diagnostic adjunct and an independent predictor of malignancy after adjustment for jaundice. However, its modest sensitivity at the conventional cutoff limits its use as a stand-alone diagnostic marker. Preoperative CA19-9 may provide exploratory prognostic stratification after resection, although the observed survival difference by CA19-9 group was not statistically significant in this cohort.

## Introduction

Pancreatic cancer remains one of the most lethal malignancies worldwide, with most patients presenting at an advanced stage and a five‑year overall survival (OS) rate below 10%. Serum carbohydrate antigen 19‑9 (CA19-9) is the most extensively studied tumor marker in pancreatic cancer. In symptomatic patients, the CA19-9 level achieves a sensitivity of approximately 79%-81% and a specificity of 82%-90% in diagnosing pancreatic carcinoma [1-3]. Interpretation of the findings, however, is limited by false‑positive elevations in cholestasis and false‑negative results among individuals who lack the Lewis antigen (approximately 5%-10% of the population) [1,4-6]. Thus, an isolated CA19-9 value should always be interpreted in terms of the clinical context and imaging results.

Despite these limitations, CA19-9 can nevertheless help distinguish malignant from benign pancreatic lesions and provide prognostic information. For example, patients with normal preoperative CA19-9 levels (<37 U/mL) generally experience longer survival (approximately 32-36 months) than those with elevated levels (>37 U/mL), whose median survival is closer to 12-15 months [1,7,8]. Very high levels (>1000 U/mL) are highly specific for pancreatic malignancy and frequently reflect advanced, unresectable disease [1-3]. Prospective trials (e.g., The Radiation Therapy Oncology Group (RTOG) 9704) have also demonstrated that the postoperative CA19-9 level is a strong prognostic marker: patients with a post-resection CA19-9 level above 90 U/mL have substantially worse survival than those with ≤90 U/mL [9-11]. Real-world surgical cohorts are particularly informative because they include the heterogeneous benign and malignant lesions encountered in routine pancreatic practice, together with common confounders such as obstructive jaundice, rather than only highly selected trial populations. We, therefore, hypothesized that in our institutional cohort, CA19-9 would independently predict malignant pathology and provide exploratory prognostic stratification after resection.

## Materials and methods

Study design and setting

This was a retrospective study conducted at Al-Latron Military Hospital, part of the Jordanian Royal Medical Services, in Amman, Jordan. After obtaining Institutional Review Board approval from the Jordanian Royal Medical Services (approval number: 2025-16-25), we identified all consecutive patients who underwent pancreatic resection at the hospital between January 1, 2016, and June 30, 2024. The start date was chosen because electronic medical records were not widely implemented across the Royal Medical Services before January 2016, whereas the end date was selected to allow adequate follow-up for all included patients at the time of analysis. The procedures included pancreaticoduodenectomy, distal pancreatectomy, and total pancreatectomy, all performed for suspected pancreatic or periampullary neoplasms.

Study population and eligibility criteria

Inclusion criteria were (1) pancreatic resection during the study period, (2) histopathological confirmation of the resected specimen, and (3) sufficient clinical records for abstraction of baseline, operative, and follow-up data. Patients without a recorded preoperative CA19-9 value remained eligible for descriptive and survival analyses when otherwise eligible but were excluded from CA19-9-based diagnostic analyses. Patients with pancreatic neuroendocrine tumors were excluded from analyses involving the CA19-9 level because these tumors do not consistently express the antigen.

A lesion was considered to have a malignant pathology if it was confirmed histopathologically to be pancreatic ductal adenocarcinoma or a periampullary malignancy (e.g., distal cholangiocarcinoma, ampullary carcinoma, or invasive intraductal papillary mucinous neoplasm), whereas benign pathologies included lesions classified as chronic pancreatitis, benign intraductal papillary mucinous neoplasms, or another non-neoplastic lesion.

Data collection and clinical variables

Demographic characteristics, clinical presentations (including jaundice), operative details, pathologic diagnoses, and follow-up details were collected from the electronic medical records.

OS was defined as the time from resection to death from any cause or last follow-up, and progression-free survival (PFS) was defined as the time from resection to recurrence or progression or last follow-up. Vital status and recurrence were ascertained from clinical records and imaging reports.

CA19-9 measurement and definitions

Serum CA19-9 levels were measured as part of standard care at the centralized laboratory of the Jordanian Royal Medical Services using a Roche Diagnostics cobas e series analyzer (Roche Diagnostics International AG, Basel, Switzerland based on electrochemiluminescence immunoassay (ECLIA) technology. The institutional upper reference limit was 37 U/mL. Because all samples were processed in the same centralized laboratory using the same platform and standard protocol, inter-laboratory assay variability was minimized.

In patients with obstructive jaundice (total bilirubin >2 mg/dL), biliary drainage (endoscopic or percutaneous) was performed prior to resection whenever feasible. When multiple CA19-9 values were available, post-drainage measurements were preferentially analyzed.

Patients were categorized according to preoperative CA19-9 levels as normal (<=37 U/mL) or elevated (>37 U/mL). Postoperative CA19-9 values, when available, were typically drawn four to eight weeks after resection and prior to initiation of adjuvant therapy; however, because the timing was not fully standardized in this retrospective cohort, postoperative observations were summarized descriptively and were not used for formal comparative modeling. Values >90 U/mL were considered high-risk based on prior trial evidence [9-11].

Study endpoints

The primary endpoint was the diagnostic performance of preoperative CA19-9 in predicting malignant versus benign pathology. Secondary endpoints included OS and PFS among patients with malignant lesions, including stratification by preoperative CA19-9 level (≤37 vs. >37 U/mL).

Statistical analysis

Continuous variables are presented as medians with interquartile ranges (IQRs) and were compared using the Mann-Whitney U test. Categorical variables were compared using the chi-square (χ²) test or Fisher’s exact test, where appropriate.

Receiver operating characteristic curves (ROCs) were generated to assess diagnostic discriminability, with areas under the curve (AUC) and 95% CIs calculated using the nonparametric DeLong method [12]. Sensitivity and specificity with 95% CIs were reported at prespecified thresholds of 20, 37, and 100 U/mL.

CA19-9 values were log10-transformed for regression analyses due to skewed distribution; undetectable values were assigned a small positive constant prior to transformation. Multivariable logistic regression was used to assess malignancy probability as a function of log10(CA19-9) and the presence of jaundice. 

No imputation was performed for missing data. Diagnostic analyses were restricted to patients with available preoperative CA19-9 measurements, and denominator values are reported for each analysis.

Survival was estimated using the Kaplan-Meier method, with comparisons performed using the log-rank test. Multivariable Cox regression was not performed due to the limited number of events. Analyses were conducted using IBM SPSS Statistics software, version 26.0 (IBM Corp., Armonk, NY, USA) and R version 4.1 (The R Core Team, R Foundation for Statistical Computing, Vienna, Austria), with a two-sided α level of 0.05 defining statistical significance.

Ethics statement

The study was approved by the Royal Medical Services Human Research Ethics Committee (approval number 2025-16-25). Given the retrospective design, a waiver of informed consent was granted

## Results

Among the 81 patients included in the study, 63 (77.8%) demonstrated a malignant pathology, and 18 (22.2%) were benign. Preoperative CA19-9 values were available for 73 patients (59 with malignant lesions, 14 with benign lesions). The groups were similar in terms of age (median 61 vs. 58 years; p=0.38) and sex distribution; jaundice was more frequent among those with malignancies (65% vs. 28%; p=0.01). Table 1 summarizes the clinicopathologic data and diagnostic metrics of the groups.

**Table 1:** Clinicopathologic comparisons and diagnostic performance Preoperative CA19-9 data were available for 59 patients with malignant lesions and 14 patients with benign lesions; the full cohort (63 malignant, 18 benign) was used for the other variables. Continuous variables are reported as the medians (IQRs). P‑values from the Mann–Whitney U (continuous) or chi-square (χ²)/Fisher’s exact (categorical) test. Statistically significant p-values are marked with an asterisk (*). IQR: interquartile range; CI: confidence interval; AUC: area under the curve; OS: overall survival; PFS: progression-free survival

Variable/Analysis	Malignant (n = 63)	Benign (n = 18)	Statistic/Effect	95% CI	p-value	Value
Age, years (median, IQR)	61 (53-70)	58 (39-64)	Mann–Whitney U	—	0.38	
Male sex, %	62	61	χ^2^	—	0.95	
Jaundice present, %	65	28	χ^2^	—	0.01*	
Pre‑operative CA19-9 (U/mL), median (IQR)^*^	86 (15–298)	12 (6–27)	Mann–Whitney U	—	0.008*	
AUC: CA19-9 for malignancy	—	—	AUC	0.71–0.90	< 0.001*	
Sensitivity at 37 U/mL	—	—	Proportion	48–74		62%
Specificity at 37 U/mL	—	—	Proportion	62–98		89%
Logistic regression: log10(CA19-9) → malignancy	—	—	OR	1.2–13.0	0.02*	3.97
Logistic regression: jaundice → malignancy	—	—	OR	0.6–2.8	0.37	1.3
One‑year OS rate: malignant vs. benign, %	93.7	100	Log‑rank	—	0.03*	
Three‑year OS rate: malignant, %	81	—	Kaplan–Meier	—	—	
1/3-year PFS rate: malignant, %	80/60	—	Kaplan–Meier	—	—	

The median preoperative CA19-9 level was 86 U/mL (IQR 15-298 U/mL) in patients with malignant lesions and 12 U/mL (IQR 6-27 U/mL) in those with benign lesions (p=0.008). The AUC for discriminating a malignant from a benign pathology was 0.82 (95% CI 0.71-0.90). At a CA19-9 level of 37 U/mL, the sensitivity was 62% (95% CI 48%-74%), and the specificity was 89% (95% CI 62%-98%). Lowering the cutoff to 20 U/mL increased the sensitivity to 70% but reduced the specificity to 78%; increasing it to 100 U/mL reduced the sensitivity to 44% but improved the specificity to 100%.

According to the logistic regression analyses, log10(CA19-9) independently predicted malignancy (OR 3.97 per 10‑fold increase in CA19-9 level; 95% CI 1.2-13.0; p=0.02), whereas jaundice did not (OR 1.3; 95% CI 0.6-2.8; p=0.37).

**Figure 1:**
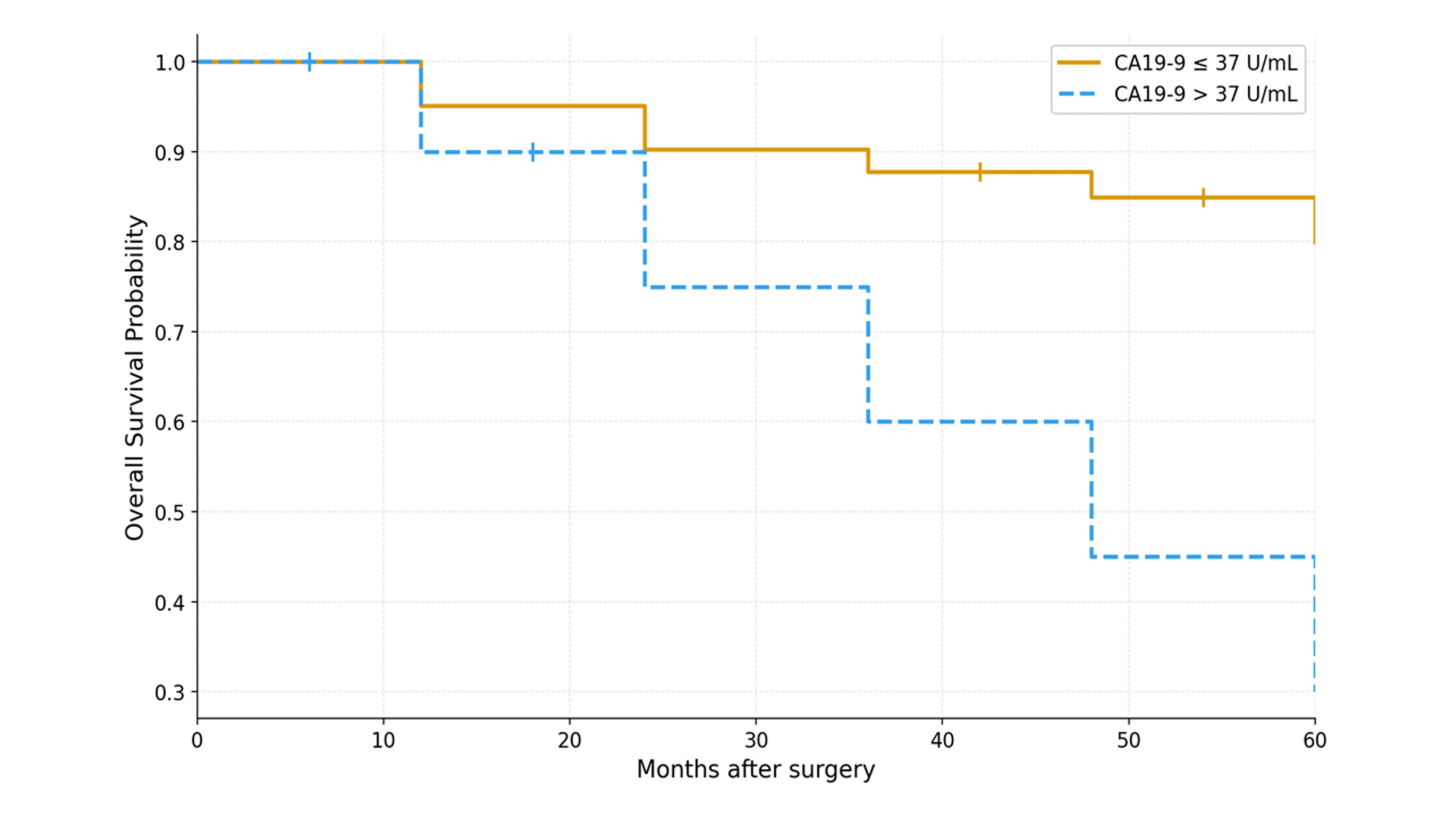
Kaplan–Meier overall survival curves for patients with malignant lesions stratified by preoperative CA19-9 level (≤37 U/mL vs. >37 U/mL). Although the curves are numerically distinct, the difference did not reach statistical significance (log‑rank p=0.15). Tick marks indicate censored observations.

**Figure 2:**
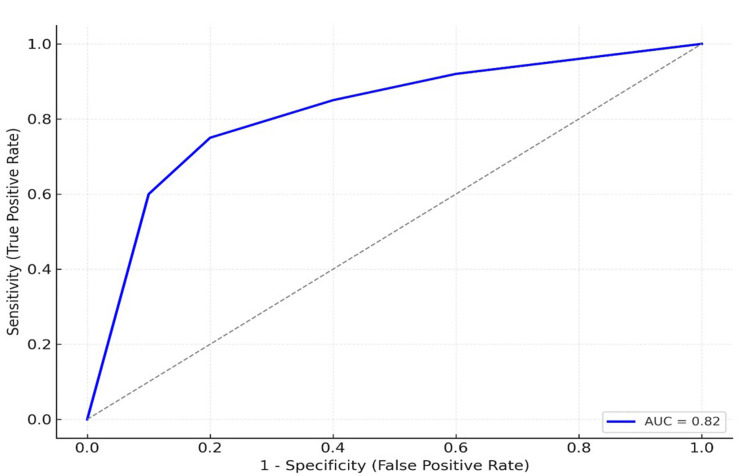
. Receiver operating characteristic (ROC) curve for preoperative CA19-9 in discriminating malignant lesions from benign lesions. Area under the curve (AUC) = 0.82 (95% CI 0.71–0.90). The 37 U/mL threshold corresponds to an ~62% sensitivity and 89% specificity in this cohort.

The median follow‑up was 24 months. For patients with malignant lesions, the one‑ and three‑year OS rates were 93.7% and 81.0%, respectively, and the median OS was not reached; 12 death events were observed during follow-up. In contrast, at the last follow-up, patients with benign lesions had a 100% survival rate. OS differed significantly between malignant and benign patients (log‑rank p = 0.03). Within the malignant lesion group, patients with a preoperative CA19-9 ≤ 37 U/mL showed a nonsignificant trend toward greater OS compared with those with > 37 U/mL (log‑rank p = 0.15). The one‑ and three‑year PFS rates for patients with malignant lesions were 80% and 60%, respectively. Among patients with available postoperative measurements, postoperative CA19-9 elevation appeared to accompany early recurrence; however, postoperative sampling was not standardized, and this observation is descriptive only. The Kaplan-Meier curves, stratified by preoperative CA19-9 level, are shown in Figure 1, while the AUC curves are shown in Figure 2.

## Discussion

In our current series, preoperative CA19-9 differentiated malignant from benign pancreatic lesions with moderate-to-good diagnostic discrimination (AUC 0.82). However, sensitivity at the conventional 37 U/mL cutoff was modest (62%) and lower than the approximately 79%-81% reported in pooled symptomatic cohorts [1-3]. High CA19-9 levels remained independently associated with malignancy after accounting for jaundice, indicating value regardless of the presence of cholestasis [13].

Cholestasis, cholangitis, and other benign hepatobiliary disorders can increase CA19-9 levels, sometimes dramatically [4,5]. Accordingly, repeating CA19-9 after biliary decompression is essential when a patient with jaundice presents with an elevated value. Emerging bilirubin-adjusted indices, such as the CA19-9/direct bilirubin (DBil) ratio, can help distinguish autoimmune pancreatitis or benign obstruction from pancreatic head cancer in jaundiced patients; recent data show that CA19-9/DBil improves discrimination in this context [14]. These practical steps reduce false positives without compromising sensitivity.

Our cohort showed numerically better outcomes among patients with normal preoperative CA19-9 (<=37 U/mL) than in those with elevated levels, although this difference was not statistically significant (p=0.15). These findings are consistent in direction with those of larger series demonstrating substantial differences in survival following stratification according to CA19-9 level [7,8,13]. Given the limited number of death events (n=5), these survival findings should be interpreted cautiously. In addition, studies involving prospective datasets (e.g., RTOG 9704) have demonstrated that failure to normalize the CA19-9 level postresection, particularly in patients with levels >90 U/mL, predicts earlier recurrence and worse survival despite adjuvant therapy [9-11]. While our postoperative sampling was not standardized, these external data support the integration of the early postresection CA19-9 level into adjuvant decision-making and surveillance frequency.

CA19-9 alone is imperfect and should complement imaging and histology rather than replace them. Combining the CA19-9 level with other serum markers (e.g., the levels of CEA and CA125) can improve diagnostic performance in the early stages for high-risk or symptomatic populations [15]. Liquid-biopsy assays, such as those involving circulating tumor DNA (ctDNA) or tumor cells (CTCs), have shown promise for improving diagnostic yields and postoperative monitoring when used alongside CA19-9 [16,17]. These approaches are not yet considered a standard of care but increasingly demonstrate incremental value in pancreatic ductal adenocarcinoma detection and surveillance [13,15,17]. In contrast, population screening for pancreatic cancer is not recommended (Grade D recommendation by the U.S. Preventive Services Task Force (USPSTF)) because of its low positive predictive value and lack of proven mortality benefit in asymptomatic adults [18].

This study has several limitations. These include its retrospective design, single-center setting, modest sample size (including eight patients with missing preoperative CA19-9 values), and lack of routine Lewis antigen typing (potentially misclassifying nonproducers as false negatives). Moreover, we did not standardize the timing of postoperative CA19-9 measurements, precluding a detailed analysis of early dynamics postresection; therefore, postoperative observations were descriptive only. Finally, tumor-type heterogeneity (e.g., inclusion of periampullary cholangiocarcinoma, ampullary carcinoma, and invasive intraductal papillary mucinous neoplasm) may attenuate marker performance for any single histology but reflects real-world indications for pancreatic resection.

For patients with suspected pancreatic cancer, we recommend (1) interpreting CA19-9 levels alongside cross-sectional imaging and the clinical context; (2) repeating CA19-9 testing after biliary decompression when jaundice is present; (3) considering bilirubin-adjusted indices (e.g., CA19-9/DBil) in persistently jaundiced patients; and (4) using the postoperative CA19-9 level, ideally obtained four to eight weeks after resection, to help guide risk stratification and surveillance when available [9-11,14]. Prospective studies should standardize assay timing (pre-/post-drainage and postresection), incorporate Lewis typing, and evaluate multimarker and liquid-biopsy strategies for additive value over CA19-9 [13,15,17].

## Conclusions

CA19‑9 can provide useful diagnostic information as an adjunct to clinical assessment and imaging when evaluating pancreatic and periampullary tumors, particularly when interpreted in the context of bilirubin status. Given its limited sensitivity at the conventional threshold, CA19‑9 should not be used in isolation to exclude malignancy. Further prospective studies with standardized postoperative sampling are needed to better define its prognostic role after resection.
